# Recreational Drug Use at a Music Festival: A Dual Approach Using Hair Biomarkers Analysis and Participant Self‐Reported Drug Use

**DOI:** 10.1002/dta.70076

**Published:** 2026-04-23

**Authors:** A. Y. Simão, T. Rosado, M. Barroso, M. Andraus, E. Gallardo

**Affiliations:** ^1^ RISE‐Health, Departamento de Ciências Médicas, Faculdade de Ciências da Saúde Universidade da Beira Interior Covilhã Portugal; ^2^ Laboratório de Fármaco‐Toxicologia, UBIMedical Universidade da Beira Interior Covilhã Portugal; ^3^ Centro Académico Clínico das Beiras (CACB)‐Grupo de Problemas Relacionados com Toxicofilias Covilhã Portugal; ^4^ AlphaBiolabs Warrington UK; ^5^ Serviço de Química e Toxicologia Forenses Instituto de Medicina Legal e Ciências Forenses ‐ Delegação do Sul Lisbon Portugal; ^6^ Cansford Laboratories Limited Cardiff UK

**Keywords:** drugs of abuse, hair analysis, LC–MS/MS, music festival, statistical analysis, survey

## Abstract

The growing prevalence of substance use and its associated health consequences highlights the need for reliable approaches to assess consumption patterns and validate self‐reported data. This study, conducted at an international music festival in Portugal, aimed to characterise substance use by integrating objective toxicological findings with self‐reported information. Quantitative hair analysis was combined with survey data from 249 participants recruited in 2022 and 2023. Hair samples were analysed by liquid chromatography–tandem mass spectrometry to detect psychoactive substances and metabolites. Self‐reported use was assessed across multiple timeframes, from same‐day consumption to use within the previous year. Alcohol (96%) and cannabinoids (90%) were the most frequently self‐reported substances overall, based on lifetime self‐reported use. Overall, 50% of participants tested positive for at least one compound in the analysed hair samples, with cocaine, MDMA and ketamine being the most commonly detected substances (24.5%, 24.1% and 22.9%, respectively). Some participants who denied consumption tested positive, particularly for MDMA and ketamine. Self‐reported non‐use was inversely associated with hair positivity for MDMA (OR = 0.25, 95% CI: 0.09–0.65) and ketamine (OR = 0.19, 95% CI: 0.09–0.40), compared with self‐reported users. Discrepancies were also observed for cannabinoids, highlighting limitations of self‐reported data. Strong correlations were identified between cocaine and benzoylecgonine, and between cannabis‐related analytes (cannabidiol and THC), supporting the consistency of the toxicological results. Integrating self‐reported data with objective biological measures improved data reliability, revealed polydrug use patterns and supported substance use monitoring in high‐risk populations such as music festival attendees in this high‐exposure festival setting.

## Graphical abstract

Combined self‐report and hair analysis in festival attendees (*n* = 249) revealed substantial discrepancies in drug use reporting, particularly for stimulants and dissociatives. This integrated approach enhances detection of poly‐drug use and provides a more reliable basis for monitoring and harm‐reduction strategies.

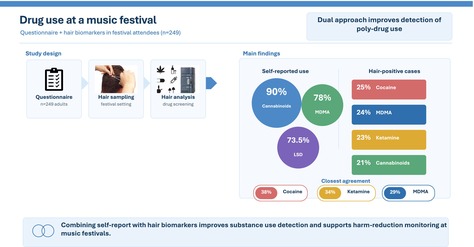

**Abbreviations**
AEME: anhydroecgonine methyl ester
BZE: benzoylecgonine
CBD: cannabidiol
CBN: cannabinol
FCT: Fundação para a Ciência e a Tecnologia
FEDER: Fundo Europeu de Desenvolvimento Regional
GHB: gamma‐hydroxybutyric acid
LC–MS/MS: liquid chromatography–tandem mass spectrometry
MDA: 3,4‐methylenedioxyamphetamine
MDMA: 3,4‐methylenedioxymethamphetamine
MRM: multiple reaction monitoring
NPS: new psychoactive substances
PCP: phencyclidine
PMMA: para‐methoxymethamphetamine
*r*
: Pearson correlation coefficient
*R* ^2^
: coefficient of determination
SPE: solid phase extraction
SPSS: Statistical Package for the Social Sciences
THC: Δ9‐tetrahydrocannabinol
THC‐COOH: 11‐nor‐9‐carboxy‐Δ9‐tetrahydrocannabinol
UHPLC: ultra‐high‐performance liquid chromatography
UPLC: ultra‐performance liquid chromatography
*χ* ^2^
: chi‐square (statistical test)

## 1 Background

The global burden of substance use, involving both licit and illicit drugs, remains a major public health concern. Music festivals, as large‐scale social and cultural events, represent distinctive contexts for recreational drug use, shaped by the music, collective atmosphere and a perceived reduction in risk. Numerous studies have shown that substance use prevalence among festival attendees is consistently higher than in the general population, positioning these events as particularly relevant settings for investigating consumption patterns and polysubstance use behaviours [1, 2, 3, 4, 5, 6, 7, 8, 9].

Beyond prevalence, research conducted in festival and electronic dance music environments has documented a broad and evolving range of substances. While recreational drugs such as cannabis and 3,4‐methylenedioxymethamphetamine (MDMA) remain prevalent, these settings are also marked by the use of stimulants, dissociative drugs and an increasing presence of novel psychoactive substances (NPS), reflecting shifts in drug markets and availability [10, 11, 12]. Longitudinal studies among nightlife and electronic dance music attendees further highlight sustained use of substances such as cocaine, amphetamine‐type stimulants and ketamine, alongside temporal fluctuations influenced by availability, perceived safety and social norms [8, 10].

In this context, hair analysis has emerged as a valuable approach for assessing drug use in festival populations. Unlike blood and urine testing, which reflect recent consumption, hair analysis provides a retrospective detection window spanning several months [13]. This allows for the assessment of chronic exposure and polysubstance use patterns, while offering practical advantages, such as non‐invasiveness and sample stability. When combined with self‐reported data, hair testing strengthens data reliability by mitigating common survey‐related biases, including recall errors and intentional underreporting or overreporting [14, 15].

Indeed, discrepancies between self‐reported substance use and analytically confirmed exposure have been repeatedly documented in nightlife and festival settings. These inconsistencies underscore the limitations of questionnaires when used alone and highlight the added value of objective toxicological measures. Increasing evidence of unintentional drug exposure, driven by substance adulteration or mislabelling, further reinforces the need to integrate biological analysis to obtain more accurate prevalence estimates [9, 12, 14].

Studies conducted in electronic dance music and festival contexts have provided deeper insight into these phenomena. For example, Palamar and Keyes [10] reported persistently high prevalence of MDMA, cocaine and ketamine use among electronic dance music party attendees, with variations linked to availability and perceived risk. Drug use in these environments is often spontaneous, increasing the likelihood of polysubstance consumption and exposure to unexpected compounds [8]. Hair‐based toxicological investigations have consistently revealed mismatches between declared and detected substances, indicating both underreporting and inadvertent intake due to adulteration [12, 14]. Moreover, analytical studies have identified NPS and unexpected compounds co‐occurring with classic recreational drugs, illustrating the complexity and unpredictability of drug use patterns in these settings [11, 12, 14].

Substance use at music festivals is commonly motivated by the desire to enhance sensory experiences, ease social bonding, and respond to peer influence. However, psychoactive substance consumption is also associated with significant risks, including acute health incidents, accidents and longer‐term mental health consequences. Polysubstance use, which is particularly prevalent in festival environments, further complicates risk assessment due to unpredictable pharmacological interactions [16, 17].

Research has also identified demographic and situational factors linked to higher‐risk drug use at festivals, such as younger age, frequent attendance and prolonged engagement in nightlife activities. Importantly, polysubstance use has been consistently associated with increased morbidity and, in severe cases, mortality, especially when combinations of central nervous system depressants and stimulants are involved [18]. These findings highlight music festivals as high‐risk environments that require targeted surveillance and harm reduction strategies.

Supporting this evidence, Palamar et al. [16] investigated rave attendance and illicit drug use among US high school seniors, demonstrating that rave attendance was relatively common and strongly associated with higher rates of illicit drug use, particularly among frequent attendees. Excluding marijuana, lysergic acid diethylamide (LSD) was the most reported substance, while ketamine and gamma‐hydroxybutyric acid (GHB) use was nearly six times more prevalent among rave attendees than non‐attendees.

Similarly, Geuens et al. [19] provided empirical data from a major Belgian music festival, identifying the festival setting as a high‐risk environment for both excessive alcohol consumption and illicit drug use. While the prevalence of classic recreational substances mirrored broader European trends, particularly high levels of MDMA and ketamine suggested event‐specific preferences or increased availability. The detection of NPS, including synthetic cathinones, reinforced the dynamic and evolving nature of drug markets. Notably, the unexpected identification of pharmaceuticals such as flecainide and amlodipine raised serious safety concerns, as unintentional consumption of these substances may have severe health consequences [19]. These findings underscore the importance of drug checking services and targeted public health interventions.

Against this background, the present study aims to investigate the prevalence and patterns of substance use among attendees at a large‐scale music festival in Portugal. By integrating quantitative hair analysis with self‐reported survey data, this research seeks to provide a comprehensive characterisation of substance use behaviours, examine concordance between reported and detected substances and explore patterns of polysubstance consumption. Overall, the study contributes to the growing evidence supporting integrative methodological approaches in substance use research, particularly in high‐risk festival populations.

## 2 Materials and Methods

### 2.1 Participants, Survey Description and Hair Collection

Participants were recruited during an international music festival held in Portugal in July 2022 and July 2023. This festival is renowned for its focus on psychedelic culture, electronic music and holistic practices. Festival attendees were approached randomly at designated research stands and invited to participate in the study. Eligibility criteria included being 18 years of age or older. Participation involved completion of an anonymous, self‐administered questionnaire, followed by the voluntary donation of a hair sample for toxicological analysis. Participation was voluntary and no financial or material incentives were provided. A total of 249 participants completed the questionnaire and provided a hair sample for toxicological analysis. As recruitment took place in an open festival setting, the total number of individuals approached was not systematically recorded; however, refusals were uncommon. Hair was collected as follows: a pencil thickness strand was collected from the posterior vertex region. In cases where hair was longer than ~5 cm, the proximal part was covered on foil and samples were stored in an envelope with a number given corresponding to the survey of the participant. In cases where hair was shorter than ~5 cm, the proximal part was not identified as 10 mg of the whole strands were analysed. No information regarding hair characteristics was recorded.

The questionnaire collected information on sociodemographic characteristics (including age, sex, education level, employment status, and country of residence) and substance use history for at least 14 substances (‘alcohol’, ‘marijuana/weed’, ‘cigarettes’, ‘ecstasy’, ‘opioids and heroin’, ‘cocaine’, ‘LSD’, ‘ketamine’, ‘methamphetamine/amphetamines’, ‘unknown powders’, ‘tranquilizers’, ‘stimulants’, ‘sedatives’ and ‘others’). For each substance, participants were asked to indicate the timeframe of last consumption (‘on the day of the survey’; ‘2–3 days ago’; ‘4–7 days ago’; ‘more than one week ago, but less than one month ago’; ‘more than one month ago, but less than one year ago’; ‘more than one year ago’).

Additional questions were designed to capture broader patterns of substance use, including typical contexts of consumption (e.g., solitary or group use location of use), substance mixing practices, perceived health effects, age of initiation and self‐perceived awareness of drug‐related risks. The questionnaire was deliberately designed to be concise to maintain participants' attention and engagement in the context of an on‐site music festival survey. Consequently, for participants reporting multiple consumption episodes or use of multiple substances, responses reflect overall patterns of use (at least within the past year) rather than individual consumption events.

The study protocol was approved by the Ethics Committee of the University of Beira Interior (ce‐UBI‐Pj‐2022‐041) and conducted in accordance with the Declaration of Helsinki.

No financial or material incentives were offered.

### 2.2 LC–MS/MS Analysis

A rapid liquid chromatography–tandem mass spectrometry (LC–MS/MS) method was used to detect and quantify a total of 82 substances, including classic drugs of abuse, their metabolites, and selected new psychoactive substances (NPS), using a targeted analytical approach (Table S1). Reference standards for quality control, calibration, and deuterated internal standards for all analytes were acquired from Cerilliant (Merck, UK), Chiron (UK) and Lipomed (LGC Standards, UK). Hair samples (10 mg) underwent two sequential washes with methanol (1 mL each) under brief agitation, followed by pulverisation. The whole hair strand was analysed without segmentation. Therefore, positive findings reflect drug incorporation over the analysed hair length rather than over a fixed and uniform timeframe across participants. Solid‐phase extraction (SPE) was conducted using HCX mixed‐mode cartridges (Biotage, UK) for classic drugs of abuse and filtration plates (Biotage, UK) for NPS. Details of the extraction protocols, instrument and validation data are proprietary. Extracted samples were evaporated to dryness and reconstituted for LC–MS/MS analysis. Chromatographic separation was achieved using a Waters Acquity UPLC HSS T3 column (1.8 μm, 3.0 × 50 mm) coupled with an Agilent 1290 Infinity UHPLC system (Agilent, UK) for traditional classic drugs and a Xevo TQ‐S micro–Triple Quadrupole mass spectrometer combined with an Acquity I‐Class UPLC system for NPS. Mass spectrometric detection used a triple quadrupole in positive ionisation mode for most analytes, except THC‐COOH, which was detected in negative ionisation mode. Mobile phases included 5 mM ammonium acetate with 0.01% formic acid for traditional classic drugs and 1% formic acid for cannabinoids. Methanol with 0.01% formic acid was used as the organic phase, while acetonitrile with 0.1% formic acid was employed for NPS. Quantitative analysis was performed using dynamic multiple reaction monitoring (MRM) mode. The method has been accredited to ISO/IEC 17025 (UKAS). The method was fully validated, with validation parameters including selectivity, specificity, linearity, precision, intra‐day and inter‐day accuracy, matrix effects and recovery. Validation of the method yielded determination coefficients (*R* ^2^) exceeding 0.98 for all analytes spiked into blank hair samples processed through the extraction protocol, suggesting a linear model for calibration. Data were analysed using IBM SPSS Statistics Version 28 and Microsoft Excel 2010. Variables were categorised as nominal, ordinal or numeric, and statistical tests were selected based on the distribution characteristics of the data.

## 3 Results and Discussion

### 3.1 Demographic Characteristics

The survey responses were collected in 2 years (July 2022 and July 2023), with 45.4% (*n* = 113) of participants responding in 2022 and 54.6% (*n* = 136) in 2023. The demographic characteristics and consumption patterns of the respondents revealed a diverse participant pool. Table S2 shows a schematic representation of the obtained data.

### 3.2 Substance Use History by Group

Percentages for self‐reported substance use were derived from any reported consumption across the questionnaire timeframes and therefore reflect overall lifetime use rather than a specific recent period. A description of the data obtained from the survey regarding the substance use history for each group of substances is summarised in Table 1.

**TABLE 1:** Self‐reported substance use by timeframe among festival attendees, % (*N*).

Substance	On day of survey	2–3 days ago	4–7 days ago	1 week to 1 month	1 month to 1 year	> 1 year ago	Yes, consumed but did not answer how long ago	Cumulative consumption percentage (all timeframe)	Never
Alcohol	32.5 (81)	47.0 (117)	9.2 (23)	2.8 (7)	1.2 (3)	1.2 (3)	2 (5)	96	4.0 (10)
Cigarettes	39.0 (97)	11.6 (29)	4.8 (12)	5.2 (13)	4.4 (11)	12.0 (30)	0.8 (2)	77.9	22.1 (55)
Cannabinoids	14.9 (37)	26.1 (65)	10.0 (25)	12.0 (30)	13.7 (34)	12.0 (30)	1.2 (3)	90	10.0 (25)
Cocaine	0.8 (2)	3.6 (9)	5.2 (13)	9.2 (23)	16.9 (42)	20.5 (51)	0.4 (1)	56.6	43.4 (108)
MDMA	0.4 (1)	—	6.4 (16)	14.5 (36)	29.7 (74)	25.7 (64)	0.8 (2)	77.5	22.5 (56)
Methamphetamine	—	0.4 (1)	0.4 (1)	1.2 (3)	4.0 (10)	17.3 (43)	0.4 (1)	23.7	76.3 (190)
Amphetamine‐type stimulants (except methamphetamine and MDMA)	0.4 (1)	2.8 (7)	1.6 (4)	6.0 (15)	6.0 (15)	15.7 (39)	67.5 (168)	32.5	—
Heroin	0.4 (1)	—	0.4 (1)	—	0.8 (2)	15.3 (38)	—	16.9	83.1 (207)
Opioids (non‐heroin)	—	0.8 (2)	1.6 (4)	1.2 (3)	5.6 (14)	21.7 (54)	—	30.9	69.1 (172)
Ketamine	0.8 (2)	2.4 (6)	3.6 (9)	12.4 (31)	16.9 (42)	18.9 (47)	0.4 (1)	55.4	44.6 (111)
LSD	0.4 (1)	0.8 (2)	2.8 (7)	10.0 (25)	24.1 (60)	34.1 (85)	1.2 (3)	73.5	26.5 (66)
Tranquilisers	0.8 (2)	0.8 (2)	1.6 (4)	2.0 (5)	4 (11)	19.7 (49)	—	29.3	70.7 (176)
Sedatives	—	—	0.8 (2)	1.2 (3)	0.8 (2)	13.3 (33)	83.5 (208)	16.5	0.4 (1)
Psilocybin	0.4 (1)	—	0.4 (1)	1.6 (4)	2.4 (6)	1.2 (3)	—	6.0	94.0 (234)
DMT/ayahuasca	—	—	0.4 (1)	0.4 (1)	0.8 (2)	0.4 (1)	0.4 (1)	2.4	97.6 (243)
Cathinones^a^	—	—	0.8 (2)	0.8 (2)	—	0.4 (1)	1.6 (4)	3.6	96.4 (240)
PCP	0.4 (1)	—	—	0.4 (1)	0.4 (1)	16.1 (40)	—	17.3	82.7 (206)
GHB	0.4 (1)	—	—	—	1.2 (3)	17.3 (43)	—	18.9	81.1 (202)
Unknown powders	0.4 (1)	0.4 (1)	0.8 (2)	0.4 (1)	1.6 (4)	16.1 (40)	0.4 (1)	20.1	79.9 (199)

^a^ Cathinones include 2‐CB, mephedrone and 3‐MMC.

Overall, self‐reported substance use was dominated by legal substances and cannabinoids, while the use of most other illicit drugs was uncommon and largely non‐recent. Alcohol was the most widely used substance, with 32.5% of participants reporting consumption on the day of the survey and 47.0% reporting use within the previous 2 to 3 days. Only 4.0% of the interviewees reported never having consumed alcohol. Cigarette use was similarly prevalent, with 39.0% reporting use on the day of the survey and 22.1% reporting never having smoked before.

Cannabinoids were the most frequently reported illicit substance and showed a notably recent pattern of use. 14.9% of participants reported use on the day of the survey, and 26.1% reported use within the previous 2 to 3 days. Use declined over a more extended period, with 12.0% reporting use within the previous month and 12.0% reporting last use more than 1 year ago. Only 10.0% of participants indicated never having used cannabinoids, highlighting their widespread and recent use within this population.

In contrast, cocaine use was considerably less common within the month preceding hair collection (18.9%). Use on the day of the survey was rare (0.8%), and recent use within 2 to 3 days was reported by only 3.6% of participants. Most participants reported either last use more than 1 year ago (20.9%) or never having used cocaine (43.4%). A similar pattern was observed for MDMA. While recent use was uncommon (0.4% on the day of the survey), a substantial proportion of participants reported use within the past year (51.0%) or more than one year ago (25.7%), suggesting moderate lifetime prevalence but limited recent use.

Regarding the use of LSD, ketamine, MDMA and cocaine, although a large proportion of participants reported having used these substances at some point, recent use (within the month preceding the survey) was substantially lower when compared with cannabis consumption.

For tranquilisers, opioids, psilocybin, amphetamine‐type stimulants (except MDMA), sedatives, heroin, PCP, GHB and unknown powders, participants also reported predominantly non‐recent use, with most indicating last consumption more than 1 month prior to survey administration.

Notably, the reported percentages of self‐reported substance use represent lifetime use, as they encompass any period covered by the questionnaire, whereas the positive hair analysis findings reflect drug incorporation across the entire hair sample analysed, resulting in a non‐standardised and participant‐dependent detection window determined by hair length.

### 3.3 Quantification Results in Real Hair Samples: Positive Cases

A total of 249 hair samples were collected in July 2022 and July 2023 and analysed for drugs of abuse, with 125 samples (50%) testing positive for at least one substance. The analytical panel included a broad range of substances commonly targeted in hair testing, including both classical drugs of abuse and several new psychoactive substances. Considering that the study was conducted at an international music festival attracting participants from different countries, a wide screening panel was used to capture a broad spectrum of potential exposures. However, the composition of the analytical panel may not fully reflect the current drug market in Portugal, and this aspect should be considered when interpreting the results.

Table S3 presents the limit of detection, the limit of quantitation and the cut‐off concentrations used to define positive cases for each compound. Among the detected compounds, benzoylecgonine (BZE) was the most frequently identified, present in 61 participants (24.5%), followed closely by MDMA, found in 60 participants (24.1%). Ketamine was identified in 57 participants (22.9%), reflecting substantial use, while cocaine was detected in 46 participants (18.5%). Cannabinoids were found in 52 participants (20.9%), underscoring the prevalence of cannabis‐related substances.

Several non‐hydrolytic metabolites were also detected, strongly suggesting drug intake rather than external contamination. Norketamine was detected in 23 participants (9.2%), and cocaethylene, a compound that forms when cocaine and alcohol are co‐ingested, was present in 21 participants (8.4%). Norcocaine, another cocaine metabolite, was detected in 17 participants (6.8%). Amphetamine was found in 10 participants (4.0%); 3,4‐methylenedioxyamphetamine (MDA), often associated with MDMA use, was identified in eight participants (3.2%).

Concerning 11‐nor‐9‐carboxy‐Δ9‐tetrahydrocannabinol (THC‐COOH), a key metabolite of cannabis, it was present in four participants (1.6%), further demonstrating cannabis metabolism.

Less frequently detected substances included anhydroecgonine methyl ester (AEME), a by‐product of cocaine consumption, found in three participants (1.2%). Methamphetamine, mescaline and *p*‐methoxymethamphetamine (PMMA) were each detected in 1 participant (0.4%), reflecting minimal use within this cohort.

In summary, BZE, MDMA and ketamine were the most frequently detected compounds, followed by cocaine and cannabinoids. The detection of metabolites such as norketamine, cocaethylene and norcocaine highlights active metabolic processing of these substances among users. Conversely, the rare detection of substances such as methamphetamine, mescaline, and PMMA suggests limited use in this population. These findings provide valuable insights into the prevalence and patterns of substance use, as well as the metabolic profiles associated with these substances. Results are shown in Table S4. In fact, these results resonate with results from Cunha et al. [20], where MDMA was similarly the most detected substance, present in 88.5% of oral fluid samples, highlighting its consistent popularity in electronic music settings. Ketamine was also the most prevalent NPS, detected in 29.4% of the samples [20]. The prominence of MDMA, amphetamines and cocaine at Hungarian festivals aligns with findings from the current study, where once again MDMA and ketamine featured prominently [21]. These substances often dominate recreational drug use patterns due to their stimulant and dissociative effects, particularly during high‐energy music events.

### 3.4 Demographic Influence on Drug Concentration in Hair Samples: A Statistical Analysis

For each substance detected in hair, a high concentration was defined when the measured concentration was equal to or above the median of the detected concentrations, whereas a low concentration was defined when the measured concentration was below the median.

Regarding gender, only one statistically significant association was observed, namely, with high cocaine concentrations determined in hair samples. Pearson's chi‐square (*χ* ^2^) test showed a significant association (*χ* ^2^[1, *N* = 46] = 5.254, *p* = 0.022). High concentrations were predominantly observed in males (87.0%), whereas no such differences were observed for low concentrations.

Regarding age, no statistically significant associations were observed between age groups and the concentrations determined in hair samples for any of the analysed compounds. Nevertheless, descriptive trends were observed. The 18–25 age group predominantly presented high ketamine concentrations (84.6%). In the intermediate age groups (26–30 and 31–35 years), ketamine concentrations were typically low (62.5% and 80.0%, respectively). In the older age groups (36–40 and 41–50 years), high ketamine concentrations were again predominantly observed (66.7% and 100.0%, respectively).

For cocaine, lower concentrations were typically observed in the younger age groups, namely 18–25 years (57.1%) and 26–30 years (63.2%). In contrast, higher cocaine concentrations were predominantly observed in the older age groups, specifically 31–35 years (66.7%), 36–40 years (83.3%) and 41–50 years (100.0%).

A similar trend was observed for MDMA, with younger age groups (18–25 years) predominantly presenting low concentrations (61.5%), whereas older age groups showed typically higher concentrations, particularly in the 31–35 (63.6%), 36–40 (66.7%) and 41–50 (100.0%) age ranges.

As samples were collected over two consecutive years (2022 and 2023), an additional statistical evaluation was performed. A statistically significant association was observed for MDMA concentrations determined in hair samples (*χ* ^2^[1, *N* = 60] = 4.033, *p* = 0.045). In 2023, MDMA concentrations were predominantly high (66.7%) when compared with those observed in 2022 (40.7%). Although no statistically significant association was observed for THC, a similar trend was identified, with 60.9% of positive samples in 2023 presenting high concentrations compared with 36.8% in 2022.

## 4 Comparison of Self‐Reported Drug Use and Quantitative Hair Analysis

One of the aims of this study was to evaluate the association between self‐reported drug use, obtained via questionnaires and objective toxicological findings in hair samples. To this end, cross‐tabulation analyses and odds ratios (ORs) were calculated using SPSS (v.29). Pearson's chi‐square (*χ* ^2^) test, or Fisher's exact test when appropriate, was used to assess statistical significance, while ORs with 95% confidence intervals (CIs) were used to estimate the strength and direction of the associations. The analysis focused on five substance groups: cannabinoids, cocaine, ketamine, ecstasy/MDMA and methamphetamine. Among the 249 participants, statistically significant associations between self‐reported use and positive hair toxicology results were observed for ketamine, MDMA and cocaine (Table 2). The strongest association was found for cocaine (OR = 7.53, 95% CI: 3.39–16.70), followed by ketamine (OR = 5.22, 95% CI: 2.49–10.92) and MDMA (OR = 4.07, 95% CI: 1.54–10.73). These findings indicate that participants reporting use of these substances had higher odds of testing positive in hair analysis.

**TABLE 2:** Odds ratios (ORs) and 95% confidence intervals (CIs) for the association between self‐reported use and positive hair toxicology results by substance group.

Substance	OR	CI lower	CI upper	*p* (*χ* ^2^)
Ketamine	5.22	2.49	10.92	< 0.001
MDMA	4.07	1.54	10.73	0.003
Canabinoids	1.06	0.38	2.98	0.909
Cocaine	7.53	3.39	16.70	< 0.001
Methamphetamine	1.02^a^	0.98	1.05	0.072

^a^ Methamphetamine OR comes from an isolated case toxicology hair positive result; therefore, this result must be interpreted carefully.

No statistically significant association was observed for cannabinoids (OR = 1.06, 95% CI: 0.38–2.98), which may reflect underreporting, misreporting, variability in consumption patterns or differences in detection windows. Methamphetamine showed extremely low analytical prevalence, with only one positive hair sample identified. Therefore, the corresponding OR should be interpreted with caution.

Complementary analyses were conducted focusing on participants who denied drug use in the questionnaire but presented a positive hair toxicology result for the same substance (Table 3). In this group, significant inverse associations were observed for cocaine (OR = 0.13, 95% CI: 0.06–0.30), ketamine (OR = 0.19, 95% CI: 0.09–0.40) and MDMA (OR = 0.25, 95% CI: 0.09–0.65), indicating lower odds of hair positivity among participants reporting non‐use. No significant associations were observed for cannabinoids or methamphetamine.

**TABLE 3:** Odds ratios (ORs) and 95% confidence intervals (CIs) for the association between self‐reported non‐use and positive hair toxicology results by substance group.

Substance	OR	IC lower	IC upper	*p* (χ^2^)
Ketamine	0.19	0.09	0.40	< 0.001
MDMA	0.25	0.09	0.65	0.003
Canabinoids	0.94	0.34	2.64	0.909
Cocaine	0.13	0.06	0.30	< 0.001
Methamphetamine	0.98^a^	0.95	1.02	0.072

^a^ Methamphetamine OR comes from an isolated case toxicology hair positive result; therefore, this result must be interpreted carefully.

Altogether, these findings highlight the limitations of relying exclusively on self‐reported data, particularly in festival settings where altered states of consciousness, social influences and variability in drug composition may affect user awareness and reporting accuracy.

Additionally, quantitative hair analysis and descriptive statistics of the detected substances are summarised in Table 4. Among the analytes quantified, cocaine presented the highest mean concentration (mean = 7.45 ng/mg), with a wide concentration range (0.60–74.50 ng/mg), indicating substantial variability among positive samples. Cocaine‐related metabolites, including BZE, norcocaine, cocaethylene and AEME, were also detected at measurable levels, with BZE showing a relatively high mean concentration (1.68 ng/mg) and a maximum value of 25.57 ng/mg.

**TABLE 4:** Summary of the descriptive statistics of quantitative hair analysis for drug detection (ng/mg).

Substance	Average (ng/mg)	Standard deviation (ng/mg)	Median (ng/mg)	Minimum (ng/mg)	Maximum (ng/mg)
Cocaine	7.45	15.16	2.25	0.60	74.50
Norcocaine	0.16	0.19	0.09	0.06	0.78
Cocaethylene	0.27	0.29	0.19	0.05	1.14
BZE	1.68	4.23	0.44	0.05	25.57
AEME	1.83	1.21	1.70	0.70	3.10
Ketamine	3.50	6.82	1.20	0.20	43.60
Norketamine	0.63	0.96	0.30	0.10	4.70
MDMA	1.95	3.63	0.70	0.20	19.70
MDA	0.46	0.38	0.35	0.20	1.30
Amphetamine	4.78	9.61	0.45	0.20	30.30
Methamphetamine	10.00	n.a.	10.00	10.00	10.00
THC	0.50	0.87	0.14	0.05	4.32
CBN	0.44	0.83	0.17	0.05	4.26
CBD	0.33	0.22	0.29	0.07	0.59
THC‐COOH	0.0029	0.0017	0.0030	0.0007	0.0048
Mescaline	2.00	n.a.	2.00	2.00	2.00
PMMA	0.30	n.a.	0.30	0.30	0.30

Abbreviation: n.a., not available.

Ketamine was another frequently detected substance, presenting a mean concentration of 3.50 ng/mg (range: 0.20–43.60 ng/mg), while its primary metabolite, norketamine, showed lower concentrations (mean = 0.63 ng/mg). For MDMA, a mean concentration of 1.95 ng/mg was observed (range: 0.20–19.70 ng/mg), accompanied by the detection of its metabolite MDA (mean = 0.46 ng/mg).

Amphetamine exhibited a relatively high mean concentration (4.78 ng/mg), with values ranging from 0.20 to 30.30 ng/mg. Methamphetamine was detected in a single hair sample at a concentration of 10.00 ng/mg; therefore, no variability measures could be calculated for this substance. Similarly, mescaline and PMMA were each detected in only one sample, with concentrations of 2.00 and 0.30 ng/mg, respectively.

Cannabinoids generally showed lower concentration levels compared to other substance groups. THC, CBN and CBD presented mean concentrations of 0.50, 0.44 and 0.33 ng/mg, respectively. THC‐COOH exhibited the lowest concentrations overall, with a mean value of 0.0029 ng/mg and a narrow concentration range (0.0007–0.0048 ng/mg), reflecting minimal incorporation into hair samples.

An analysis of agreement responses according to substance use history revealed marked differences between participants who reported consumption and those who did not. For all substances evaluated, agreement was consistently higher among non‐consumers (Table 5).

**TABLE 5:** Agreement between self‐reported substance use and hair toxicology results.

Substance	Self‐reported use (*N*)	Agree *N* (%)	Disagree *N* (%)	Self‐reported non‐use (*N*)	Agree *N* (%)	Disagree *N* (%)
Methamphetamine	59	1 (1.7)	58 (98.3)	190	190 (100)	0 (0)
Ketamine	138	47 (34.1)	91 (65.9)	111	101 (91.0)	10 (9.0)
Cocaine	141	53 (37.6)	88 (62.4)	108	100 (92.6)	8 (7.4)
MDMA	193	55 (28.5)	138 (71.5)	56	51 (91.1)	5 (8.9)
Cannabis	224	47 (21.0)	177 (79.0)	25	20 (80.0)	5 (20.0)

*Note:* Agreement refers to a positive hair result among participants reporting substance use, and to a negative hair result among participants reporting no substance use.

Methamphetamine use was associated with a very low level of agreement, with only 1.7% of consumers reporting agreement, compared to 100% among non‐consumers. Similarly, consumers of ketamine and cocaine showed lower agreement rates (34.1% and 37.6%, respectively) than non‐consumers (91.0% and 92.6%, respectively).

For MDMA and cannabis, agreement among consumers was observed in 28.5% and 21.0% of cases, respectively, whereas agreement among non‐consumers exceeded 80% for both substances. Overall, these findings suggest a consistent association between substance consumption and lower levels of agreement with the evaluated outcome.

The low level of agreement between self‐reported substance use and hair sample results may be partially explained by discrepancies between the reported time frame of consumption and the hair length analysed, which may not have covered the period during which consumption occurred.

When the analysis was restricted to participants reporting substance use within the previous year, agreement with hair toxicology results remained low across all substance groups (Table 6). Methamphetamine showed the highest concordance, although this finding should be interpreted cautiously due to the small number of positive cases. For ketamine, cocaine, and MDMA, agreement was observed in only a small minority of self‐reported past‐year users, while no concordant cases were identified for cannabinoids. These findings suggest that self‐reported past‐year use does not necessarily correspond to a positive hair result, likely reflecting differences in frequency and intensity of use, variability in drug incorporation into hair and the absence of a standardised analysed hair length covering a comparable retrospective window across participants.

**TABLE 6:** Agreement between self‐reported substance use for past‐year ago and hair toxicology results.

Substance	Self‐reported use (*N*)	Agree *N* (%)	Disagree *N* (%)
Methamphetamine	43	13 (30.2)	30 (69.8)
Ketamine	47	2 (4.3)	45 (95.7)
Cocaine	51	3 (5.9)	48 (94.1)
MDMA	64	3 (4.7)	61 (95.3)
Cannabis	30	0 (0)	30 (100)

*Note:* Agreement refers to a positive hair result among participants reporting substance use in the past year.

Overall, these findings highlight the value of integrating objective methodologies, such as hair analysis, to complement self‐reported data. Although some level of alignment was observed for certain substances, the marked discrepancies identified for others, particularly cannabinoids, reinforce the need for objective measures to validate self‐reported behaviours. The chi‐square analysis provided additional insight into the relationship between reported consumption and toxicological findings, while also underscoring the importance of combining subjective and objective approaches to improve the reliability and validity of substance use research.

### 4.1 Analysis of Substance Consumption Patterns: Polydrug Use Patterns and Correlation Between Substances

To assess whether the detected substances were associated with one another, a Pearson correlation analysis was performed. This analysis aimed to identify significant relationships between substances detected in hair samples, considering the strength of the correlation coefficients.

The Pearson correlation coefficients (*r*) were interpreted as follows:

- *r* ≥ 0.75: indicated a very strong positive correlation
- 0.50 ≤ *r* < 0.75: indicated a strong positive correlation.
- 0.25 ≤ *r* < 0.50: indicated a moderate positive correlation.
- *r* < 0.25: indicated a weak positive correlation.

A strong positive correlation was observed between ketamine and norketamine (*r* = 0.585, *p* < 0.001), which is expected given that norketamine is a metabolite of ketamine. Similarly, BZE and cocaine showed a very strong positive correlation (*r* = 0.836, *p* < 0.001), supporting BZE as a reliable metabolite marker of cocaine use. CBN and THC also exhibited a very strong positive correlation (*r* = 0.800, *p* < 0.001), consistent with their common origin in cannabis exposure.

In addition, ketamine and cocaine showed a strong positive correlation (*r* = 0.579, *p* < 0.001), suggesting possible co‐occurrence of these substances among some participants. Cocaine and amphetamine‐type stimulants were moderately correlated (*r* = 0.477, *p* < 0.001), while THC and CBD showed a weak‐to‐moderate positive correlation (*r* = 0.264, p < 0.001), which may reflect differences in cannabis product composition and patterns of use (results are depicted in Table 7).

**TABLE 7:** Correlation analysis between psychoactive substances under study.

Substances	Pearson correlation coefficient (*r*)	*p*
Ketamine and norketamine	0.585	< 0.001
CBN and THC	0.800	< 0.001
BZE and cocaine	0.836	< 0.001
THC and CBD	0.264	< 0.001
Ketamine and cocaine	0.579	< 0.001
Cocaine and amphetamine‐type stimulants	0.477	< 0.001

## 5 Conclusions

This study underscores the value of integrating self‐reported data with quantitative hair analysis to obtain a comprehensive understanding of substance use behaviours in music festival settings. Survey responses indicated high levels of polydrug use, with alcohol and cannabinoids being the most frequently self‐reported substances and the most reported for use within the previous month. In contrast, hair analysis revealed that cocaine, MDMA and ketamine were the most frequently detected compounds, followed by cannabinoids.

The comparison between subjective and objective measures identified substantial discrepancies, with more than 62% of participants who reported substance use testing negative in hair analysis. Despite this overall low level of agreement, the highest concordance rates were observed for cocaine (37.6%), ketamine (34.1%) and MDMA (28.5%), whereas agreement for cannabinoids was notably weaker. These findings highlight the limitations of relying exclusively on self‐reported data, particularly in recreational settings characterised by recall bias, misperception and variability in analytical detection windows. Strong associations between parent drugs and their respective metabolites, as well as between cannabis‐related analytes, further supported the internal consistency and interpretative reliability of the toxicological findings.

Overall, this research provides robust evidence that combining questionnaire‐based self‐reported data with quantitative hair toxicological analysis offers a more comprehensive and reliable assessment of substance use behaviours in music festival settings. While questionnaires allow the capture of contextual information, consumption patterns and perceived behaviours, hair analysis contributes as an objective measure of cumulative exposure over extended timeframes. The integration of these complementary approaches enables the identification of discrepancies, improves the interpretation of self‐reported data and facilitates the detection of concealed or unintentional drug use. Collectively, this combined methodology enhances the understanding of polydrug use dynamics and supports the development of more effective public health surveillance, harm reduction strategies and targeted interventions in high‐risk environments.

## Author Contributions

**A. Y. Simão:** conceptualisation, formal analysis, investigation, writing – original draft; **T. Rosado:** formal analysis, writing – review and editing; **M. Barroso:** conceptualisation, methodology, project administration, supervision, writing – review and editing; **M. Andraus:** conceptualisation, methodology, project administration, supervision, writing – review and editing; **E. Gallardo:** conceptualisation, methodology, project administration, supervision, writing – review and editing.

## Funding

The authors acknowledge the financial support provided by national funds from Fundação para a Ciência e a Tecnologia (FCT) and by the European Regional Development Fund (FEDER), within the framework of PORTUGAL 2020 and the Centro Regional Operational Programme (CENTRO 2020), under the projects with references https://doi.org/10.54499/UIDB/00709/2020 and https://doi.org/10.54499/UIDP/00709/2020. This work was also supported by national funds through FCT – Fundação para a Ciência e a Tecnologia, I.P., under the project RISE‐Health (UID/06397/2025). Ana Y. Simão also acknowledges the PhD fellowship granted by FCT (reference: https://doi.org/10.54499/2020.09070.BD).

## Conflicts of Interest

The authors declare no conflicts of interest.

## Supporting information




**Table S1:** Drugs and metabolites included in hair toxicological analysis.

**Table S2:** Demographic characteristics and substance consumption patterns of survey participants (*N* = 249).

**Table S3:** Limit of detection (LOD), limit of quantification (LOQ), and cut‐off concentrations (ng/mg) adopted for the identification and interpretation of the analysed compounds.

**Table S4:** Compounds identified and concentrations of abused substances.

## Data Availability

The data that supports the findings of this study are available in the Supporting Information of this article.
