# Supplementary material for: Recreational Drug Use at a Music Festival: A Dual Approach Using Hair Biomarkers Analysis and Participant Self‐Reported Drug Use
Source: Drug Test Anal. 2026 Apr 23;18(7):831–40. doi: 10.1002/dta.70076 (PMC13327157; doi:10.1002/dta.70076)
Supplement: Supplementary file 1 — Table S1: Drugs and metabolites included in hair toxicological analysis. [file DTA-18-831-s001.docx]

# Supplementary table 1. Drugs and metabolites included in hair toxicological analysis.

| Class | Substances analysed |
| --- | --- |
| Cannabinoids (natural and synthetic) | 11-nor-Δ9-THC-carboxylic acid, THC, cannabidiol, cannabinol, CP-47,497, AM2201, AM2233, JWH-015, JWH-018, JWH-019, JWH-073, JWH-081, JWH-122, JWH-200, JWH-203, JWH-210, JWH-250, AB-FUBINACA, APINACA, AB-PINACA, PB-22, RCS-4, RCS-8, UR-144, THJ-2201, 5F-MDMB-PINACA, MDMB-CHIMICA |
| Stimulants (amphetamine-type and cocaine-related) | amphetamine, methamphetamine, cathine (norpseudoephedrine), cathinone, methcathinone, fluoromethcathinone, alpha-PVP, MDPV, mephedrone, methylone, butylone, ethylone, pentylone, methedrone, cocaine, benzoylecgonine, cocaethylene, norcocaine, anhydroecgonine methyl ester |
| Entactogens / MDMA-related compounds | MDMA, MDA, MDEA, MBDB |
| Opioids and opioid-related compounds | heroin, morphine, codeine, dihydrocodeine, oxycodone, 6-acetylmorphine, 6-acetylcodeine, fentanyl, acetylfentanyl, fluorofentanyl, alfentanil, remifentanil, pethidine, buprenorphine, norbuprenorphine, methadone, EDDP, propoxyphene, norpropoxyphene, tramadol, o-desmethyltramadol |
| Hallucinogens and dissociatives | LSD, ketamine, norketamine, PCP, mescaline, PMA, PMMA |
| NBOMe compounds | 25C-NBOMe, 25D-NBOMe, 25H-NBOMe, 25I-NBOMe |
