# Supplementary material for: Recreational Drug Use at a Music Festival: A Dual Approach Using Hair Biomarkers Analysis and Participant Self‐Reported Drug Use
Source: Drug Test Anal. 2026 Apr 23;18(7):831–40. doi: 10.1002/dta.70076 (PMC13327157; doi:10.1002/dta.70076)
Supplement: Supplementary file 2 — Table S2: Demographic characteristics and substance consumption patterns of survey participants (N = 249). [file DTA-18-831-s002.docx]

Supplementary table 2 – Demographic characteristics and substance consumption patterns of survey participants (N = 249).

| **Variable** | **Category** | **N (%)** |
| --- | --- | --- |
| Year of survey/response | 2022 | 113 (45.4) |
|  | 2023 | 136 (54.6) |
| Sex | Male | 153 (61.4) |
|  | Female | 88 (35.3) |
|  | Other | 4 (1.6) |
|  | Unknown | 4 (1.6) |
| Age group (years) | 18–25 | 48 (19.3) |
|  | 26–30 | 95 (38.2) |
|  | 31–35 | 54 (21.7) |
|  | 36–40 | 26 (10.4) |
|  | 41–50 | 9 (3.6) |
|  | >50 | 1 (0.4) |
|  | Not disclosed | 16 (6.4) |
| Sexual orientation | Heterosexual | 97 (39.0) |
|  | Bisexual | 13 (5.2) |
|  | Homosexual | 2 (0.8) |
|  | Other | 5 (2.0) |
|  | Not disclosed | 132 (53.0) |
| Marital status | Single | 192 (77.1) |
|  | Married | 42 (16.9) |
|  | Divorced/Separated | 7 (2.8) |
|  | Widowed | 1 (0.4) |
|  | Not disclosed | 7 (2.8) |
| Ethnicity | White | 215 (86.3) |
|  | Latino | 11 (4.4) |
|  | Asian | 9 (3.6) |
|  | Black | 1 (0.4) |
|  | Other | 9 (3.6) |
|  | Not disclosed | 4 (1.6) |
| Religion | None | 156 (62.7) |
|  | Christian | 36 (14.5) |
|  | Jewish | 9 (3.6) |
|  | Buddhist | 6 (2.4) |
|  | Hindu | 5 (2.0) |
|  | Not disclosed | 21 (8.4) |
| Education level | High school or less | 56 (22.5) |
|  | Bachelor’s degree | 81 (32.5) |
|  | Master’s degree | 82 (32.9) |
|  | PhD | 12 (4.8) |
|  | Not disclosed | 17 (6.8) |
| Employment status | Full-time | 141 (56.6) |
|  | Part-time | 48 (19.3) |
|  | Unemployed | 42 (16.9) |
|  | Not disclosed | 18 (7.2) |
| Residence: Continent | Europe | 180 (72.3) |
|  | Oceania | 11 (4.4) |
|  | America | 9 (3.6) |
|  | Asia | 8 (3.2) |
|  | Not disclosed | 41 (16.5) |
| Residence: Country (largest groups) | Portugal | 33 (13.3) |
|  | Germany | 30 (10.0) |
|  | United Kingdom | 26 (10.4) |
|  | France | 25 (10.0) |
|  | Belgium | 12 (4.8) |
|  | Netherlands | 11 (4.4) |
|  | Other countries | 52 (20.9) |
|  | Not disclosed | 39 (15.7) |
| Consumption behaviour: Usual consumption setting | Group | 186 (74.7) |
|  | Alone | 12 (4.8) |
|  | Both | 17 (6.8) |
|  | Not specified | 34 (13.7) |
| Consumption behaviour: Place of consumption | Home | 58 (23.3) |
|  | Parties/bars/discos | 57 (22.9) |
|  | Festivals | 38 (15.3) |
|  | Multiple locations | 27 (10.8) |
| Mixing substances | Yes | 159 (63.9) |
|  | No | 63 (25.3) |
|  | Not disclosed | 27 (10.8) |
| Number of susbtances mixed | Two | 94 (37.8) |
|  | Three | 50 (20.1) |
|  | More than three | 16 (6.4) |
| Health effects: felt unwell after use | Yes | 136 (54.6) |
|  | No | 86 (34.5) |
|  | Not disclosed | 27 (10.8) |
| Health effects: Hospitalisation | Yes | 8 (5.9) |
|  | No | 128 (94.1) |
| First substance used | Alcohol | 120 (48.2) |
|  | Cannabis | 71 (28.5) |
|  | Cigarettes | 6 (2.4) |
|  | Other | 52 (20.9) |
| Perceived awareness | Fully informed | 114 (45.8) |
|  | Sufficiently informed | 81 (32.5) |
|  | Limited awareness | 22 (8.8) |
|  | Not informed | 2 (0.8) |
|  | Not disclosed | 30 (12.0) |
