# Supplementary material for: Recreational Drug Use at a Music Festival: A Dual Approach Using Hair Biomarkers Analysis and Participant Self‐Reported Drug Use
Source: Drug Test Anal. 2026 Apr 23;18(7):831–40. doi: 10.1002/dta.70076 (PMC13327157; doi:10.1002/dta.70076)
Supplement: Supplementary file 3 — Table S3: Limit of detection (LOD), limit of quantification (LOQ), and cut‐off concentrations (ng/mg) adopted for the identification and interpretation of the analysed compounds. [file DTA-18-831-s003.docx]

**Supplementary table 3 –** Limit of detection (LOD), limit of quantification (LOQ), and cut-off concentrations (ng/mg) adopted for the identification and interpretation of the analysed compounds.

| Compound | LOD (ng/mg) | LOQ (ng/mg) | Cut-off (ng/mg) |
| --- | --- | --- | --- |
| 11-nor-Δ9-THC-carboxylic acid (THC-COOH) | 0.00051 | 0.00067 | 0.0003 |
| delta9-tetrahydrocannabinol (THC) | 0.0117 | 0.0155 | 0.05 |
| Cannabidiol (CBD) | 0.0117 | 0.0155 | 0.05 |
| Cannabinol (CBN) | 0.0117 | 0.0155 | 0.05 |
| CP-47,497 | 0.005 | 0.008 | 0.01 |
| AM2201 | 0.005 | 0.008 | 0.01 |
| AM2233 | 0.005 | 0.008 | 0.01 |
| JWH-015 | 0.005 | 0.008 | 0.01 |
| JWH-018 | 0.005 | 0.008 | 0.01 |
| JWH-019 | 0.005 | 0.008 | 0.01 |
| JWH-073 | 0.005 | 0.008 | 0.01 |
| JWH-081 | 0.005 | 0.008 | 0.01 |
| JWH-122 | 0.005 | 0.008 | 0.01 |
| JWH-200 | 0.005 | 0.008 | 0.01 |
| JWH-203 | 0.005 | 0.008 | 0.01 |
| JWH-210 | 0.005 | 0.008 | 0.01 |
| JWH-250 | 0.005 | 0.008 | 0.01 |
| AB-FUBINACA | 0.005 | 0.008 | 0.01 |
| APINACA | 0.005 | 0.008 | 0.01 |
| AB-PINACA | 0.005 | 0.008 | 0.01 |
| PB-22 | 0.005 | 0.008 | 0.01 |
| RCS-4 | 0.005 | 0.008 | 0.01 |
| RCS-8 | 0.005 | 0.008 | 0.01 |
| UR-144 | 0.005 | 0.008 | 0.01 |
| THJ-2201 | 0.005 | 0.008 | 0.01 |
| 5F-MDMB-PINACA | 0.005 | 0.008 | 0.01 |
| MDMB-CHIMICA | 0.005 | 0.008 | 0.01 |
| amphetamine | 0.084 | 0.167 | 0.2 |
| methamphetamine | 0.084 | 0.167 | 0.2 |
| cathine (norpseudoephedrine) | 0.027 | 0.050 | 0.2 |
| cathinone | 0.169 | 0.333 | 0.2 |
| methcathinone | 0.084 | 0.167 | 0.2 |
| fluoromethcathinone | 0.005 | 0.008 | 0.01 |
| alpha-PVP | 0.005 | 0.008 | 0.01 |
| MDPV | 0.005 | 0.008 | 0.01 |
| mephedrone | 0.084 | 0.167 | 0.2 |
| methylone | 0.005 | 0.008 | 0.01 |
| butylone | 0.005 | 0.008 | 0.01 |
| ethylone | 0.005 | 0.008 | 0.01 |
| pentylone | 0.005 | 0.008 | 0.01 |
| methedrone | 0.005 | 0.008 | 0.01 |
| cocaine | 0.031 | 0.082 | 0.5 |
| Benzoylecgonine (BZE) | 0.020 | 0.052 | 0.05 |
| cocaethylene | 0.020 | 0.052 | 0.05 |
| norcocaine | 0.020 | 0.052 | 0.05 |
| anhydroecgonine methyl ester | 0.031 | 0.082 | 0.5 |
| MDMA | 0.084 | 0.167 | 0.2 |
| MDA | 0.084 | 0.167 | 0.2 |
| MDEA | 0.084 | 0.167 | 0.2 |
| MBDB | 0.084 | 0.167 | 0.2 |
| heroin | 0.091 | 0.169 | 0.2 |
| morphine | 0.091 | 0.169 | 0.2 |
| codeine | 0.091 | 0.169 | 0.2 |
| dihydrocodeine | 0.091 | 0.169 | 0.2 |
| oxycodone | 0.091 | 0.169 | 0.2 |
| 6-acetylmorphine | 0.086 | 0.160 | 0.2 |
| 6-acetylcodeine | 0.086 | 0.160 | 0.2 |
| fentanyl | 0.008 | 0.015 | 0.02 |
| acetylfentanyl | 0.012 | 0.022 | 0.02 |
| fluorofentanyl | 0.010 | 0.018 | 0.02 |
| alfentanil | 0.010 | 0.018 | 0.02 |
| remifentanil | 0.011 | 0.020 | 0.02 |
| pethidine | 0.091 | 0.169 | 0.2 |
| buprenorphine | 0.008 | 0.015 | 0.01 |
| norbuprenorphine | 0.008 | 0.015 | 0.01 |
| methadone | 0.091 | 0.169 | 0.2 |
| EDDP | 0.008 | 0.015 | 0.02 |
| propoxyphene | 0.091 | 0.169 | 0.2 |
| norpropoxyphene | 0.091 | 0.169 | 0.2 |
| tramadol | 0.091 | 0.169 | 0.2 |
| o-desmethyltramadol | 0.091 | 0.169 | 0.2 |
| LSD | 0.001 | 0.003 | 0.05 |
| ketamine | 0.050 | 0.160 | 0.2 |
| norketamine | 0.050 | 0.160 | 0.2 |
| PCP | 0.050 | 0.160 | 0.2 |
| mescaline | 0.018 | 0.034 | 0.2 |
| PMA | 0.005 | 0.008 | 0.01 |
| PMMA | 0.005 | 0.008 | 0.01 |
| 25C-NBOMe | 0.005 | 0.008 | 0.01 |
| 25D-NBOMe | 0.005 | 0.008 | 0.01 |
| 25H-NBOMe | 0.005 | 0.008 | 0.01 |
| 25I-NBOMe | 0.005 | 0.008 | 0.01 |
